# Supplementary material for: A UMLS-based spell checker for natural language processing in vaccine safety
Source: BMC Med Inform Decis Mak. 2007 Feb 12;7:3. doi: 10.1186/1472-6947-7-3 (PMC1805499; doi:10.1186/1472-6947-7-3)
Supplement: Additional file 13 — RAP application directory. Sets up directory for RAP (RDF application for PHP) source code [file 1472-6947-7-3-S13.gz › rap/doc/tests/test_StoringModelsInDatabase.php.txt]

Test Store Models in Database

php
// Include RAP
define("RDFAPI\_INCLUDE\_DIR", "./../api/");
include(RDFAPI\_INCLUDE\_DIR . "RDFAPI.php");
## 1. Connect to MsAccess database (via ODBC)
## ------------------------------------------
// Connect to MsAccess (rdf\_db DSN) database using connection settings
// defined in constants.php :
$rdf\_database = ModelFactory::getDbStore();
## 2. Store a memory model in database.
## ------------------------------------
// Load an RDF-Documtent into a memory model
// Filename of an RDF document
$base="example1.rdf";
// Create a new memory model
$memModel = ModelFactory::getDefaultModel();
// Load and parse document
$memModel-load($base);
// Now store the model in database
// An unique modelURI will be generated
$rdf\_database->putModel($memModel);
// You can also provide an URI for the model to be stored
$modelURI = "example1.rdf";
// But then you must check if there already is a model with the same modelURI
// otherwise the method putModel() will return FALSE
if ($rdf\_database->modelExists($modelURI))
echo "Model with the same URI: '$modelURI' already exists";
else
$rdf\_database->putModel($memModel, $modelURI);
## 3. Create a new database model
## ------------------------------
$modelURI = "newDbModel";
// Base URI of the new model (optional)
$baseURI = "baseURIofMyNewDbModel#";
// get a new DbModel
if ($rdf\_database->modelExists($modelURI))
echo "Model with the same URI: '$modelURI' already exists";
else
$dbModel = $rdf\_database->getNewModel($modelURI, $baseURI);
## 4. List all models stored in database
## -------------------------------------
// Get an array with modelURI and baseURI of all models stored in rdf database
$list = $rdf\_database->listModels();
// Show the database contents
foreach ($list as $model) {
echo "modelURI: " .$model['modelURI'] ."  
";
echo "baseURI : " .$model['baseURI'] ."  
  
";
}
?>
